# Regulation of Free Fatty Acid Receptor 4 on Inflammatory Gene Induced by LPS in Large Yellow Croaker (*Larimichthys crocea*)

**DOI:** 10.3389/fimmu.2021.703914

**Published:** 2021-06-10

**Authors:** Mengjiao Wu, Qingfei Li, Kangsen Mai, Qinghui Ai

**Affiliations:** ^1^ Key Laboratory of Aquaculture Nutrition and Feed (Ministry of Agriculture and Rural Affairs), The Key Laboratory of Mariculture (Ministry of Education), Ocean University of China, Qingdao, China; ^2^ Laboratory for Marine Fisheries Science and Food Production Processes, Qingdao National Laboratory for Marine Science and Technology, Qingdao, China

**Keywords:** large yellow croaker, FFAR4, inflammation, macrophage, PPARγ

## Abstract

Free fatty acid receptor 4 (FFAR4) plays a key role in regulating the inflammatory response in mammals. The present study aimed to investigate the function of large yellow croaker FFAR4 on inflammation. In the present study, *ffar4* was widely expressed in 10 tissues of large yellow croaker including gill, head kidney and spleen. Further studies showed that treatment of head kidney macrophages with agonists (TUG891 or GSK137647A) or overexpression of *ffar4* reduced the mRNA expression of pro-inflammatory genes induced by LPS, and increased the expression of *pparγ*. Treatment of macrophages with antagonist AH7614 increased the mRNA expression of pro-inflammatory genes induced by LPS, and decreased the mRNA expression of *pparγ*. In order to verify the immunomodulatory effect of PPARγ, PPARγ was overexpressed in macrophages which significantly reduced the mRNA expression of pro-inflammatory genes *il6*, *il1β*, *il8*, *tnfα* and *cox2*. Moreover, results of dual-luciferase assays showed that PPARγ downregulated the transcriptional activity of *il6* and *il1β* promoters. In conclusion, FFAR4 showed anti-inflammatory effects on LPS-induced inflammation in large yellow croaker.

## Introduction

Long-chain fatty acids, especially Omega-3 fatty acids, have been shown to play an important role in inhibiting inflammatory processes (1). Over the past decade, researchers have found that Omega-3 fatty acids exert robust anti-inflammatory effects by activating G protein-coupled receptors (2). FFAR4, also known as GPR120, is one of G protein-coupled receptors (3).

FFAR4 was initially identified as an orphan receptor in the human genome database in 2003 (4). FFAR4 is a lipid-sensing receptor which has seven transmembrane domains (3). FFAR4 is highly expressed in the macrophages, which played a crucial role in anti-inflammatory (5). Omega-3 fatty acids are natural ligands of FFAR4. Several Omega-3 fatty acids such as docosahexaenoic acid (DHA) and eicosapentaenoic acid (EPA) exert their anti-inflammatory effect through FFAR4 (6, 7). DHA treatment of RAW264.7 cells for 24 h inhibited the expression of COX-2 induced by LPS, and knockdown of FFAR4 eliminated the effect of DHA, indicating that FFAR4 mediated the anti-inflammatory effect of DHA by regulating the expression of COX2 (8). In addition, a recent study pointed that activation of FFAR4 in mice protected the liver from injury *via* PPARγ which is a transcription factor that regulates inflammation and insulin sensitivity (9). However, most of the research is limited to mammals and there is no research on anti-inflammatory mechanism of FFAR4 in fish.

Large yellow croaker (*Larimichthys crocea*) is an important economically fish in China (10). Meanwhile, large yellow croaker is a good research object for studying the mechanism of inflammation (11). The purpose of this study is to clarify the mechanism of fish FFAR4 on macrophage inflammation. These insights will help us develop nutritional strategies that target FFAR4 to alleviate fish inflammation and to facilitate the healthy development of large yellow croaker breeding industry.

## Materials and Methods

### RNA Extraction, and cDNA Synthesis

All experiments conducted on fish followed the Management Rule of Laboratory Animals (Chinese Order No.676 of the State Council). Fish were anesthetized to minimize the pain before being dissected.

In the present study, tissues of large yellow croaker including gill, brain, eye, spleen, intestine, liver, muscle, head kidney, heart, and adipose tissues were collected for RNA extraction and RT-qPCR. The entire RNA extraction process used TRIzol reagent (Takara, Japan). The total RNA extraction process was carried out according to the instructions. After adjusting the RNA concentration, Prime Script-RT reagent Kit (Takara, Japan) reverse transcription kit was used to reverse transcription of the extracted RNA to synthesize the first strand of cDNA.

### Real-Time Quantitative PCR (RT-qPCR)

We used a real-time quantitative PCR instrument (Mastercycler^®^ ep realplex, Eppendorf, German) to detect the expression levels of corresponding genes in large yellow croaker and analyzed the data. The primers used in this study were shown in **Table 1**. The β-actin gene was an internal reference gene. Before the experiment officially started, the designed primer sequence was verified for amplification efficiency. The amplification efficiency is approximately in the range of 0.9-1.1, indicating that the primer can be used. Finally, we used the 2^-ΔΔCt^ method to calculate the relative expression of each gene in large yellow croaker tissues and cells (12).

**Table 1:** Primer sequences for cloning and RT-PCR.

Primer name	Sequence	Accession number
FFAR4-RT-F	GAGTCAGCCAGCAGTTGAGT	MW468049.1
FFAR4-RT-R	ACAAGGACCAGAAGAGTGCG	MW468049.1
PPARγ-RT-F	TGTCCGAGCTGGAAGACAAC	XM_010731330.2
PPARγ-RT-R	TGGGGTCATAGGGCATACCA	XM_010731330.2
IL-1β-RT-F	CATAGGGATGGGGACAACGA	XP_010734853.1
IL-1β-RT-R	AGGGGACGGACACAAGGGTA	XP_010734853.1
IL-6-RT-F	CGACACACCCACTATTTACAAC	XM_010734753.3
IL-6-RT-R	TCCCATTTTCTGAACTGCCTCT	XM_010734753.3
TNFα-RT-F	ACACCTCTCAGCCACAGGAT	EU931626.1
TNFα-RT-R	CCGTGTCCCACTCCATAGTT	EU931626.1
IFNh-RT-F	TCAGACCTCCGCACCATCA	KM501500.2
IFNh-RT-R	GCAACCATTGTAACGCCACTTA	KM501500.2
IL-8-RT-F	AATCTTCGTCGCCTCCATTGT	XM_010737667.3
IL-8-RT-R	GAGGGATGATCTCCACCTTCG	XM_010737667.3
IL-10-RT-F	AGTCGGTTACTTTCTGTGGTG	XM010738826
IL-10-RT-R	TGTATGACGCAATATGGTCTG	XM010738826
COX2-RT-F	GACACGACTTCGGAGGAGAG	KP259877
COX2-RT-R	AGACTTTGTCAGAAGTTCTTTTTGT	KP259877
FFAR4-GFP-F	GGATCCACTAGTCCAGTGTGGTGGAATGGATATTAACCGTCACAGTA	MW468049.1
FFAR4-GFP-R	GCCACTGTGCTGGATATCTGCAGAAACCCCTGCCACAAGGTGCT	MW468049.1
PPARγ-pCS2-F	CGATTCGAATTCAAGGCCTCTCGAG ATGCAAACACCAGGCAGAG	XM_010731330.2
PPARγ-pCS2-R	CTCACTATAGTTCTAGAGGCTCGAGCTAATACAAGTCCTTTATGA	XM_010731330.2
β-actin-RT-F	GACCTGACAGACTACCTCATG	GU584189
β-actin-RT-R	AGTTGAAGGTGGTCTCGTGGA	GU584189

### Cell Culture, Treatment and Transfection

Large yellow croaker head kidney macrophages were cultured in an incubator at 25°C. The medium used for culturing macrophages of large yellow croaker is DMEM/F12. HEK293T cells were cultured in a 37°C incubator. The medium used for culturing HEK293T cells is DMEM with high glucose.

The FFAR4 agonists GSK137647A (MedChemExpress, USA), TUG891 (MedChemExpress, USA) and FFAR4 antagonist AH7614 (APE*BIO, USA) were dissolved with DMSO to a concentration of 50 μM, and then diluted in DMEM/F12 to a working concentration of 20 μM. Large yellow croaker macrophages were seeded in 12-well plates. After 24 hours of culture, LPS and the agonist (GSK137647A or TUG891) or antagonist AH7614 were co-incubated with macrophages for 12 hours, and then the cells were collected for RT-PCR. The working concentration of LPS was 50 μg/mL.

The large yellow croaker FFAR4 was cloned into the pcDNA3.1-EGFP vector (Invitrogen). The primers designed were shown in **Table 1**. The open reading frame of FFAR4 with deletion of the stop condon was inserted into pcDNA3.1-EGFP plasmid (Promega, USA) using ClonExpress II One Step Cloning Kit (Vazyme Biotech, China). Plasmid DNA was extracted using EasyPure HiPure Plasmid MiniPrep Kit (TransGen Biotech, China).

The genes were overexpressed in large yellow croaker macrophages through electrotransduction. Before electroporation, large yellow croaker macrophages were cultured in serum-containing medium without penicillin-streptomycin solution. 25μl OPTI-MEM, 1×10^6^ cells and 5μg of the pcDNA3.1-EGFP-FFAR4 plasmid which was to be overexpressed formed an electroporation system. Then, the mix was added to the electroporation cup for electroporation. After 24 hours of electroporation, the gene expression was observed under fluorescence microscope and the cells were subjected to LPS (Sigma, USA). After 12 hours of LPS incubation, the cells were lysed and RNA was extracted. Then, the mRNA expression of genes related to inflammation was detected.

### Plasmid Construction and Dual-Luciferase Reporter Assay

The large yellow croaker *pparγ *was cloned into the pCS2 + vector (Invitrogen) (**Table 1**). The IL1β promoter plasmid (pGL6-IL1β) and IL6 promoter plasmid (pGL6-IL6) were stored in our laboratory. The sequences of IL1β promoter and IL6 promoter were shown in **Supplementary Figures 1 **and** 2**, respectively.

In dual-luciferase reporter assay, the activity of firefly luciferase reporter gene (pGL6-IL1β and pGL6-IL6) was detected by using luciferin as substrate, and then the activity of renilla luciferasel reporter gene was detected by using coelenterazine as substrate when the firefly luciferase was quenched. The ratio of the two luciferases was the reporter promoter activity. Before the luciferase assay, HEK293T cells were seeded into 24-well plates. We used EZ Trans Plus cell transfection reagent (LIFE iLAB BIO, China) to transfect the reporter plasmids (pGL6-IL1β, pGL6-IL6), transcription factor plasmids (pCS2-PPARγ), and pRT-TK renilla luciferase plasmids into HEK293T cells. The activity of the firefly luciferase reporter gene and the renilla luciferase reporter gene were measured on the microplate reader using TransDetect^®^ Double-Luciferase Reporter Assay Kit (Transgene Biotech, China). The ratio of the two readings was the fluorescence value after correcting the number of cells. The experiment was repeated four times.

### Statistical Analysis

The data analysis software used in this experiment was SPSS 18.0. Independent *t*-tests and the Tukey’s test were used on the data. All data were presented as mean ± SEM (standard error of the mean). When *P* < 0.05, the difference was statistically significant.

## Results

### Tissue Distribution of Large Yellow Croaker *ffar4*

**Figure 1:**
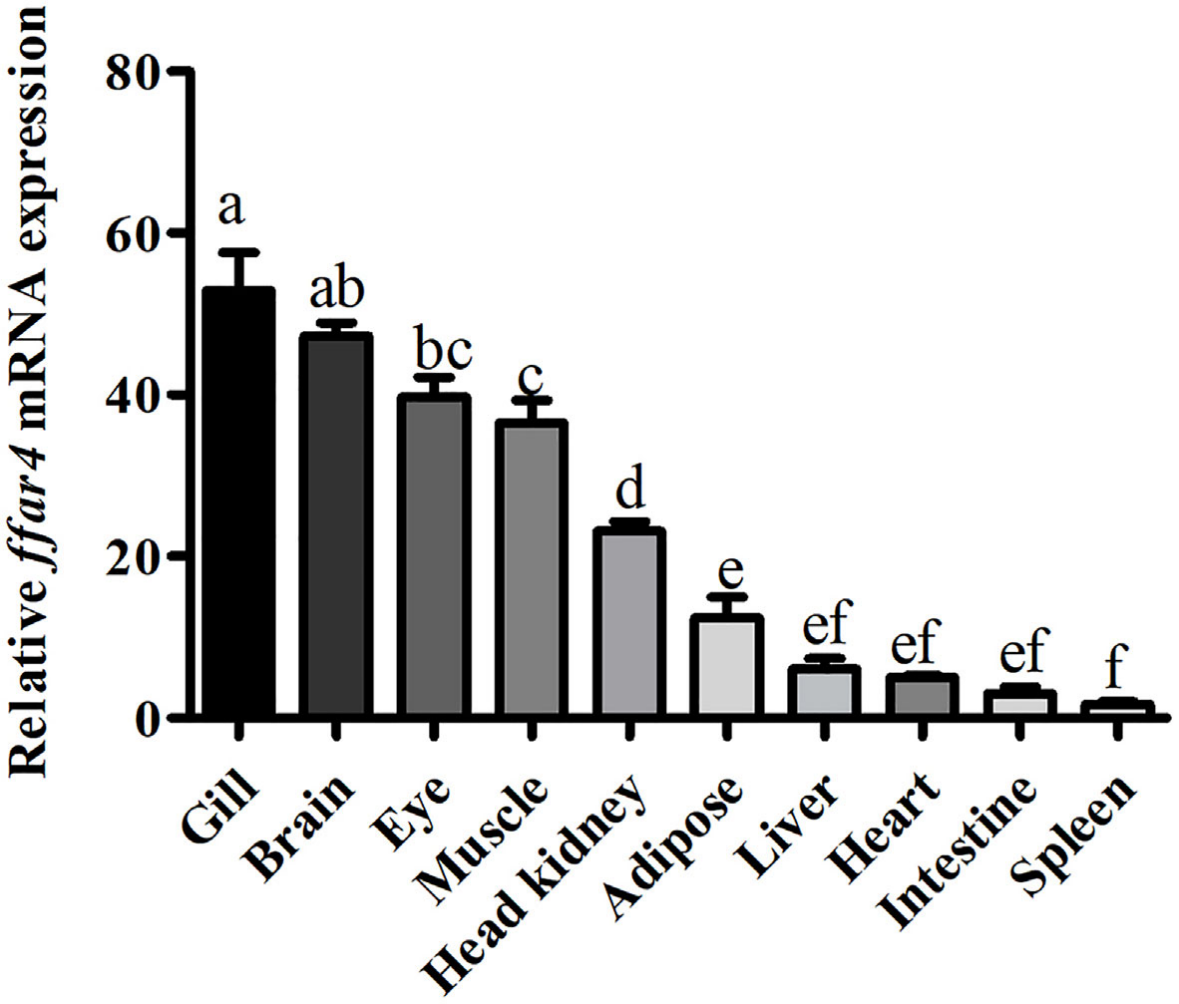
Tissue expression of *ffar4* in large yellow croaker. Data were shown as mean ± S.E.M. (n = 4). The same superscript letter means not significantly different, *P* > 0.05. Values in bars bearing the same letters were not significantly different (*P > *0.05).

The mRNA expression of *ffar4* was determined in different tissues of large yellow croaker, such as the gill, brain, eye, muscle, head kidney, adipose, liver, heart, intestine and spleen tissues (**Figure 1**).

### TUG891 or GSK137647A-Mediated Activation of FFAR4 Suppressed the LPS-Induced Expression of Pro-Inflammatory Genes

**Figure 2:**
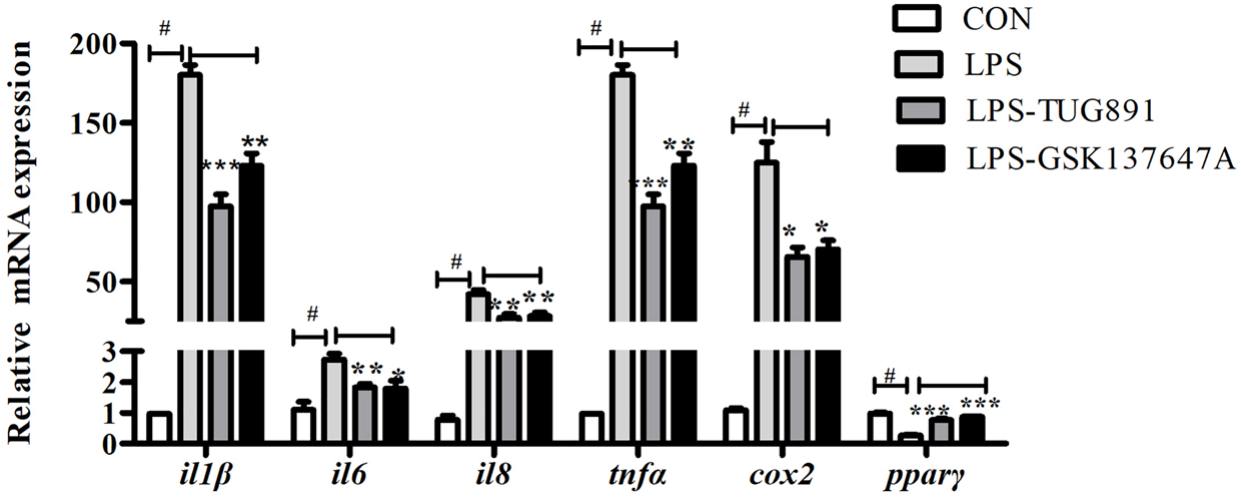
Effects of FFAR4 agonist TUG891 or GSK137647A on the expression of LPS-induced *il1β, il6, il8*, *tnfα*, *cox2* and *pparγ* in macrophages of large yellow croaker. Data were shown as mean ± S.E.M. (n = 4). ^#^P < 0.05, *P < 0.05, **P < 0.01, ***P < 0.001.

In order to study the role of FFAR4 in the regulation of LPS-induced inflammation, *in vitro* studies were conducted in macrophages of large yellow croaker. LPS significantly increased the mRNA expression of *il1β*, *il6*, *il8*, *tnfα* and *cox2*, and significantly decreased the mRNA expression of *pparγ* in macrophages (*P* < 0.05) (**Figure 2**). TUG891 or GSK137647A treatment significantly decreased LPS-induced expression of *il1β*, *il6*, *il8*, *tnfα* and *cox2* (*P* < 0.05) (**Figure 2**). TUG891 or GSK137647A significantly upregulated the mRNA expression of *pparγ* relative to the LPS treatment (*P* < 0.001) (**Figure 2**).

### AH7614-Mediated Inhibition of FFAR4 Increased LPS-Induced Pro-Inflammatory Cytokine Expression and Decreased the Expression of *pparγ*

**Figure 3:**
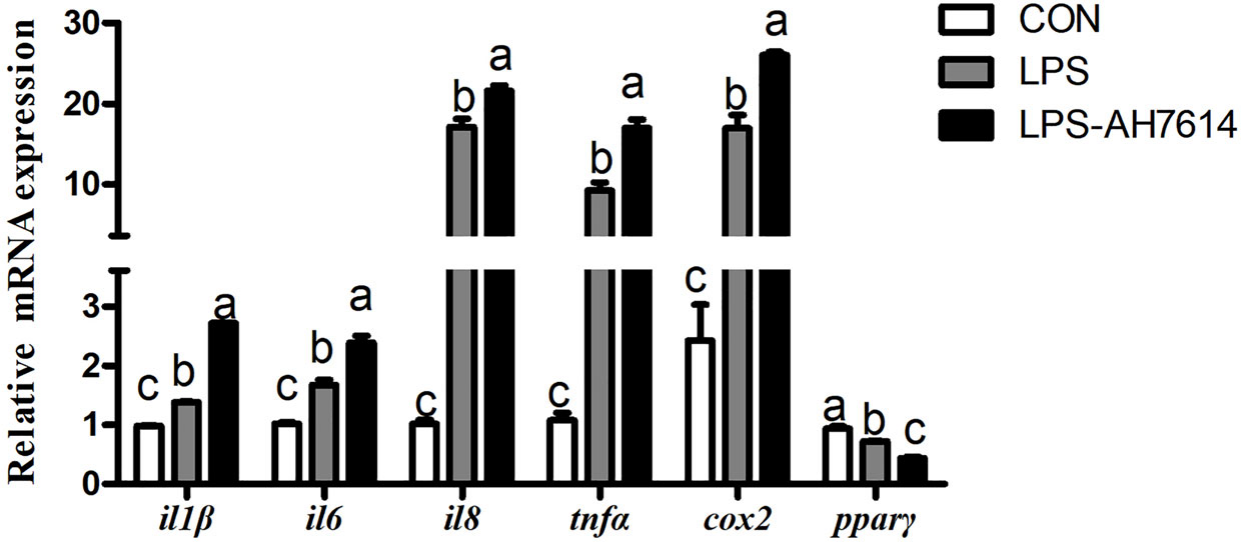
Effects of FFAR4 antagonist AH7614 on the expression of inflammation related genes induced by LPS in macrophages of large yellow croaker. Data were shown as mean ± S.E.M. (n = 4). For each gene, values in bars bearing the same letters were not significantly different among treatments (*P > *0.05).

LPS significantly increased the mRNA expression of *il1β*, *il6*, *il8*, *tnfα* and *cox2*, and significantly decreased the mRNA expression of *pparγ* in macrophages (*P* < 0.05) (**Figure 3**). AH7614 significantly up-regulated the mRNA expression of *il1β*, *il6*, *il8*, *tnfα* and *cox2*, and significantly down-regulated the mRNA expression of *ppar*γ relative to the LPS treatment (*P* < 0.001) (**Figure 3**).

### Overexpression of *ffar4* Suppressed LPS-Induced Pro-Inflammatory Cytokine Expression and Improved the Expression of *pparγ*

**Figure 4:**
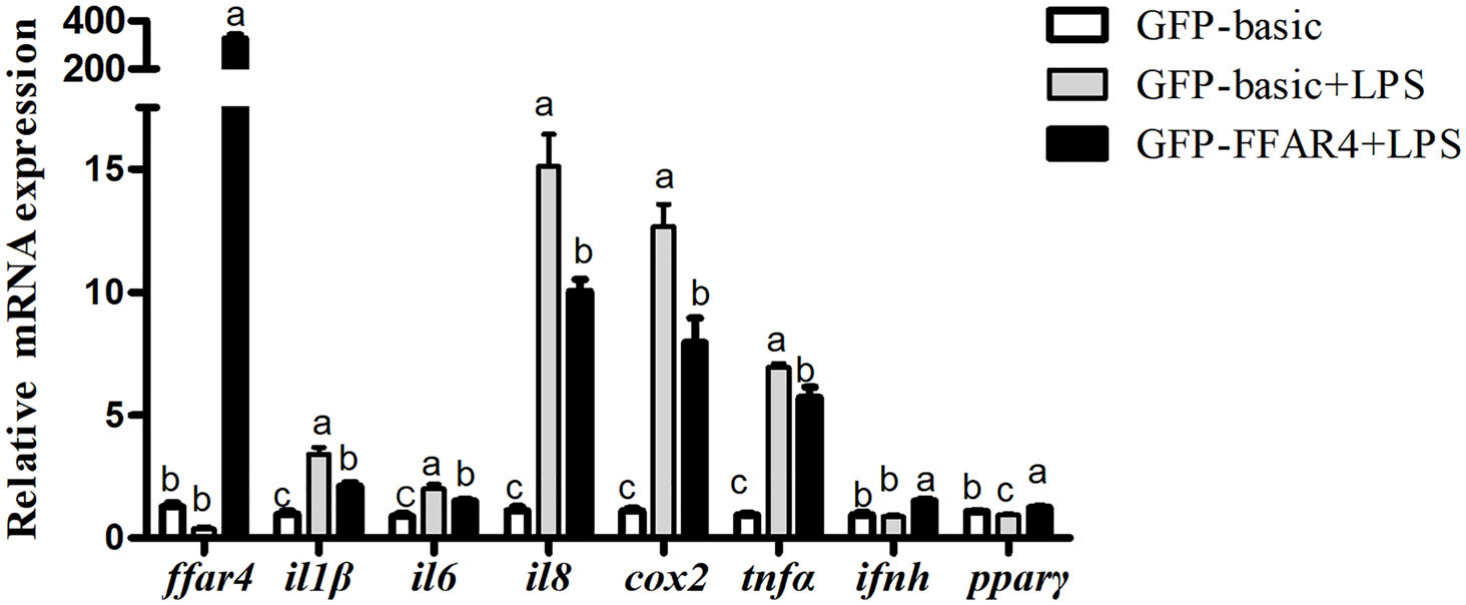
Effects of FFAR4 overexpression on LPS-induced expression of *il1β*, *il6*, *il8*, *cox2*, *tnfα*, *ifnh* and *ppar*γ in macrophages of large yellow croakers. Values were shown as mean ± S.E.M. (n = 4). For each gene, values in bars bearing the same letters were not significantly different among treatments (*P >* 0.05).

FFAR4 and GFP were overexpressed and the overexpression efficiency of FFAR4 and GFP was same under fluorescence microscope (**Supplementary Figure 3**). Following exposure to LPS, the mRNA expressions of *il1β*, *il6*, *il8*, *cox2* and *tnfα* increased significantly relative to the control group (*P* < 0.05) (**Figure 4**). However, LPS treatment significantly decreased the mRNA expressions of *ffar4* compared with the control group (*P* < 0.05) (**Figure 4**). Overexpression of *ffar4* significantly improved the expression of *pparγ* and *ifnh*, and significantly decreased the expressions of *il1β, il6, il8, cox2 and tnfα* compared to the LPS group (*P* < 0.05) (**Figure 4**).

### Overexpression of *pparγ* Suppressed LPS-Induced Pro-Inflammatory Cytokine Expression

**Figure 5:**
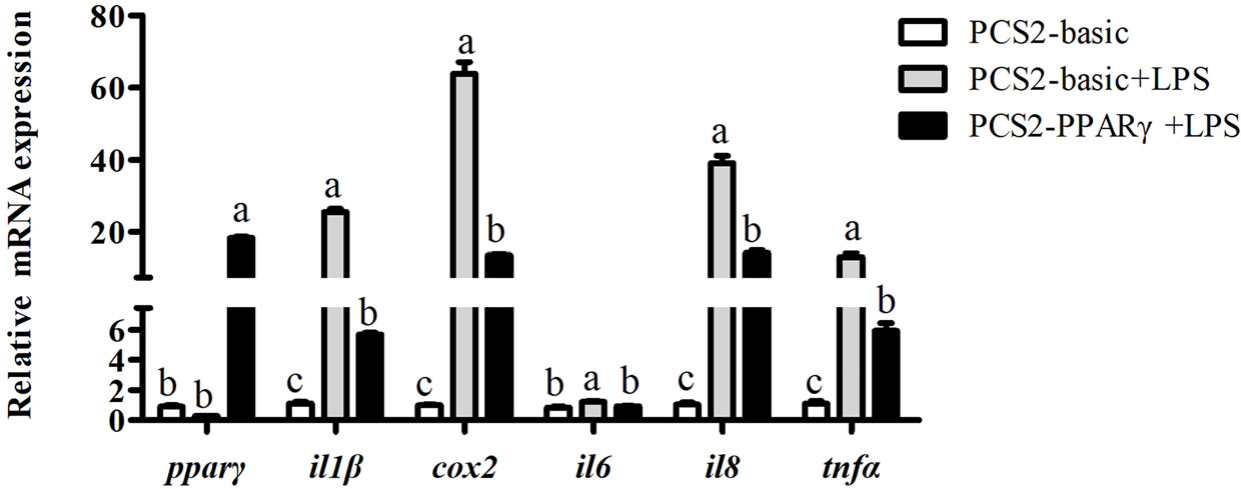
Effects of PPARγ overexpression on LPS-induced expression of *il1β*, *cox2*, *il6*, *il8*, and *tnfα* in macrophages of large yellow croakers. Values are presented as mean ± S.E.M. (n = 4). For each gene, values in bars bearing the same letters were not significantly different among treatments (*P >* 0.05).

Results above showed that *pparγ* expression of the LPS group was significantly lower than the control group, and was significantly increased by the overexpression of *ffar4*. To investigate whether the expression of *pparγ* is related to inflammatory response in large yellow croaker, the macrophages was treated with LPS and *pparγ* was overexpressed. Consistent with the previous results, LPS significantly increased the mRNA expressions of *il1β*, *cox2*, il6, *il8* and *tnfα* relative to the control group (*P* < 0.05) (**Figure 5**). Overexpression of *pparγ* significantly decreased the expressions of *il1β*, *cox2*, il6, *il8* and *tnfα* compared to the LPS group (*P*< 0.05) (**Figure 5**).

### The Regulatory Effect of PPARγ on the IL1β or IL6 Promoter

**Figure 6:**
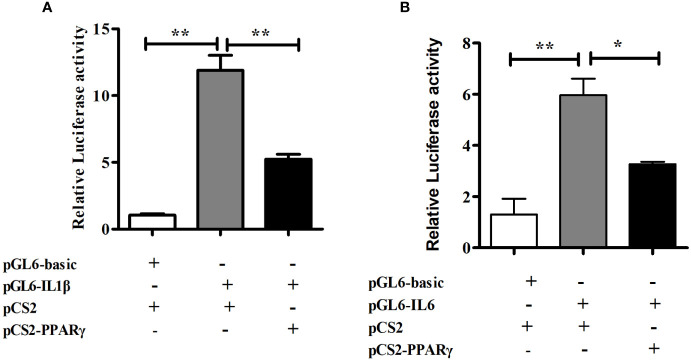
Dual-luciferase analysis of overexpression of PPARγ on large yellow croaker IL1β **(A)** and IL6 **(B)** promoter activity in HEK293T cells. PRL-CMV and pGL6-Basic used as control. Data are presented as means ± S.E.M. (n = 4). *P < 0.05, **P < 0.01.

To determine the role of PPARγ in regulating transcriptional activity of pro-inflammatory genes, HEK293T cells were co-transfected with the IL1β or IL6 promoter luciferase reporter plasmid and the PPARγ expression plasmid, using PGL6-Basic and PCS2+ as controls in the dual-luciferase reporter assay. The transcription factor PPARγ significantly down-regulated the promoter activity of IL1β (*P*< 0.01) and IL6 (*P*< 0.05) (**Figure 6**).

## Discussion

As a receptor for Omega-3 fatty acids, FFAR4 has many important functions in humans and other mammals. Many studies have shown that FFAR4 plays an important role in the treatment of diabetes (13), obesity (14), liver damage (9) and other immune diseases (15). However, there is almost no research on fish FFAR4.

In the present study, *ffar4* was widely expressed in 10 tissues of large yellow croaker, which is consistent with mammalian (5). In mammals, the main function of FFAR4 is anti-inflammatory in immune organs and cells (16). Gill, head kidney and spleen are important immune organs of fish. Therefore, the expression of FFAR4 in gill, head kidney and spleen of large yellow croaker suggests that FFAR4 may be involved in regulating inflammation in large yellow croaker. Among them, gill is not only immune organ of fish, but also the taste-sensing organ with the distribution of taste bud cells (17, 18). Studies in mammals have found that FFAR4 plays a taste perception role in taste bud cells (19, 20), so the high expression of FFAR4 in gill may also be related to taste perception. Compared with gills, FFAR4 has lower expression in head kidney and spleen. However, the function of the FFAR4 is not only affected by quantity, but also by activity. After being activated by the ligand, FFAR4 expands the signal transmission step by step by regulating the generation of the second messenger, thereby completing the cell response (21). Thus, FFAR4 can exert a powerful immune effect even in small amounts. Head kidney is a unique immune organ of fish and plays a vital role in preventing the invasion of pathogens and maintaining the health of the body in large yellow croaker (22). So next we studied the function of FFAR4 in inflammation in head kidney macrophages of large yellow croaker.

Researches show that FFAR4 played an important role in regulating inflammation in macrophages of mammals (23, 24). In order to explore whether FFAR4 is involved in inflammatory regulation in large yellow croaker head kidney macrophages, we used LPS to induce an inflammation model of large yellow croaker macrophages. In this experiment, the co-incubation of FFAR4 agonist TUG891 or GSK137647A with LPS effectively alleviated the LPS-induced inflammatory response of large yellow croaker macrophages, and significantly reduced the expression of pro-inflammatory genes *il1β, il6*, *il8*, *cox2*, and *tnfα*. In addition, overexpression of *ffar4* in large yellow croaker macrophages also inhibited inflammatory response induced by LPS. In contrast, the treatment of antagonist AH7614 aggravated the LPS-induced inflammatory response. Our results are consistent with previous findings (8, 9). Recently, some researches showed that FFAR4 can induce a significant decrease in pro-inflammatory cytokines, such as *il1β (* 16)*, il6* (25), *tnfα* (26) and *cox-2* (8). Among them, COX-2 is a prostaglandin synthesis enzyme which has been proved that the increase of COX-2 contributed to the inflammatory response (27). COX-2 derived prostaglandins are involved in a variety of inflammatory diseases (28). Therefore, the result of the down-regulation of *cox2* and other pro-inflammatory genes by FFAR4 indicates that FFAR4 is involved in the inhibition of inflammation. In addition, FFAR4 overexpression also significantly increased the expression of *ifnh*, which has not been reported in mammals. IFNh is commonly regarded as important inflammatory cytokines involved in immune responses. Studies of mammals and fish reported that IFNh pathways was triggered in the inflammatory state to protect the host (29–31). Therefore, the increase of *ifnh* after FFAR4 overexpression may be related to the regulation of immune response.

Furthermore, an interesting result we observed was that both in the FFAR4 agonist incubation experiment or the *ffar4* overexpression experiment, the expression of *pparγ* increased compared to the control group. Recently, many studies have confirmed the connection between FFAR4 and PPARγ (32). In 3T3-L1 adipocytes, FFAR4 promoted expression of *pparγ* through the Ca^2+^ and ERK1/2 signaling pathways to promote adipogenesis (33). In the Gillian’s study, fish oil prevented liver injury by normalizing the expression of *pparγ* in a FFAR4-dependent manner (9). PPARγ has been well studied in mammals for its role in immune regulation (34, 35), but there is no related report in large yellow croaker. Therefore, in order to verify that the expression of *pparγ* also exerts anti-inflammatory effects in large yellow croaker macrophages, we studied the effects of overexpression of *pparγ* on the LPS-induced inflammation of large yellow croaker. The results showed that overexpression of *pparγ* inhibited the expression of inflammatory genes induced by LPS, indicating that PPARγ indeed played a role in regulating immunity in large yellow croaker macrophages. PPARγ is a transcription factor that has been shown to regulate the expression of many inflammatory genes (36, 37). Thus, we speculated that large yellow croaker PPARγ regulates the expression of inflammatory genes by affecting promoter activity. As the largest group of cytokines, interleukins has an important function on host immune response (38). Interleukin-1 beta (IL1β) and interleukin-6(IL6) are important and representative interleukins which play crucial role in immune response (39, 40). Inflammation is often accompanied by an increase in IL1β and IL6 expression in large yellow croaker (41). However, the transcriptional regulation of PPARγ on IL1β and IL6 has not been reported in large yellow croaker. So dual-luciferase assays were carried out to study the effect of PPARγ on IL1β and IL6 promoter activity. The results of dual-luciferase assay proved that PPARγ can inhibit the activity of IL1β and IL6 promoters. In short, these results suggest that PPARγ may inhibit inflammation by suppressing IL1β and IL6 transcriptional activity.

## Conclusion

In conclusion, the immunomodulatory function of FFAR4 is conserved in large yellow croaker macrophages. Activation or overexpression of *ffar4* can reduce the mRNA expression of pro-inflammatory genes induced by LPS and increase the mRNA expression of *pparγ* which inhibits the transcriptional activity of pro-inflammatory genes. Inhibition of FFAR4 aggravated the LPS-induced inflammatory response and decreased the mRNA expression of *pparγ*. Regarding the specific signal pathways which FFAR4 regulates and how to regulate inflammation through PPARγ, further experiments are needed. Our results provide new ideas and strategies for alleviating the inflammatory response of large yellow croaker, and enrich the basic research on nutritional immunology of marine fish FFAR4.

## Data Availability Statement

The original contributions presented in the study are included in the article/**Supplementary Material**. Further inquiries can be directed to the corresponding author.

## Ethics Statement

The animal study was reviewed and approved by the Committee on the Ethics of Animal Experiments of Ocean University of China.

## Author Contributions 

QA and MW designed the experiment. MW conducted the research. QL cloned FFAR4 gene, and constructed the plasmids. MW analyzed the data. MW wrote the manuscript. KM and QA revised the article. All authors contributed to the article and approved the submitted version.

## Funding

This study was funded by the Key Program of National Natural Science Foundation of China (grant number 31830103); the National Science Fund for Distinguished Young Scholars of China (grant number 31525024).

## Conflict of Interest

The authors declare that the research was conducted in the absence of any commercial or financial relationships that could be construed as a potential conflict of interest.
