# Supplementary material for: Regulation of Free Fatty Acid Receptor 4 on Inflammatory Gene Induced by LPS in Large Yellow Croaker (Larimichthys crocea)
Source: Front Immunol. 2021 Jun 10;12:703914. doi: 10.3389/fimmu.2021.703914 (PMC8222784; doi:10.3389/fimmu.2021.703914)
Supplement: Supplementary file 1 [file DataSheet_1.pdf]

```
1       GGAGCCGTTC TTCACTCACA GTGGTCAATA GCTCTCAAAA CAACCTTGCA CCATTGGTTT
61      AATACATATT TTATCAATTT ATAACCTACT TATTTATATC TCTCACCAAT TCACATATTG
121     GTAACTTATT TAGCCTACAT TTCACCATCA ATCTACACTG CAATTAGAAA CTTATTCACA
181     ATATACACAA TTCACATATT AACACAATTC ACATATTAAC TTTTTTTTTT TTTACATCCC
241     ATCTATTTAC ACTGTTATCT GCATCACTTA TTTACATCTC ACACCATGAA TTCACATGGT
301     TAATTACATC TGACACCATA AATCTACATC AATCAAATCA ATTGTTTGCT AAACTTTCTT
361     ACATGTCACA ACATCAAACC AAATATCAGC AGTTGCCAGT CATTACAATA ATTCACACTG
421     TCTACATGCT ATATGGTCTG CTGACCTCCG GGTTTAGCGC TTCTTAACAG TTAGTTAACT
481     GAATGACATT TTATTTTATG TTTCTGTGAC TCTGATACTT TTTCCCATAA GCTTACATTG
541     TGAAAAAAGC AGCTGTAAAA TGGTGTATAT GTTTTTTAGA GCATCAAACC CCCTGTGAAA
601     TATCTTTTTT TTTTATTAGA TAGATTAGAT ATTAGATTTT TTTAGGATTC CAGAATAAAT
661     AAAAATAAAT AAAAAAACAT TAAAAAGACA ATATCTTAAT ATTCATTATA TGAAATTGTT
721     AAATGCATAT TGTTATATTA CAGTATATGC GAAATATTCC TAAACACTGT TACAATAAAA
781     ATGCAGAAGT ACAGGATTTC ATCATCCCAC TTCATGCTGA ACGGAAAATC CCATGTGATG
841     TGTATATAAC CTGCAAATCT CCACAGAGGG GCAGAACAAC ACTGACAGAA CAACTGAAGG
901     GATCTTAACC AAAGACACTA ATACAATTCC TGATCTACTG ACCTTAACAG GTAAACGCAC
961     TTCACTCTTT TTCTTTCTTT TTTTTTCTTT TTTTTTTGAA CAGATTTTGA ACAATCTTTG
1021    CTTTGGGCCA TTTTAACGGT AACACTGAGC GTAGTTTGAG ACGTGATATG ATTATAGGAT
1081    AGCTTCTTTA CTCTTCTGCC TGTCATGTTA TTAAATATGG CAGTTTTTCT AGATCATTGT
1141    GTGTTTTTAA TCTCCATCTC ATTCAATTAG TAATTAATTA ATTGAAAAAA AAGTGTTATG
1201    AATTACTGTA GTAGTAGATA GAAGTACTAT AGAAGTAAAA ATGTTAAATC ATTTTATTTC
1261    ATGAAATTAA AAAGTGTAAA ATTGAAATTC TAGCTGTATT TCCTTAATTG TTTCCTGATT
1321    CGGTGACACT GAAAATGACT CCCCTGTCTT CCCTGCGGAT CTCTTT
```

**Supplementary Figure 1.** Sequence of large yellow croaker IL1β promoter cloned. The IL1β promoter plasmid (pGL6-IL1β) contains a 1384bp promoter sequence upstream of the IL1β initiation codon(ATG).

```
1       TAGCTTCACT ATCAAGGCAG CAAGACAAAA CTAATTCCCT GACAAACTCC AACATTATGA
61      GAAAATATCT AATATCAAAG TTTTTATTCT TTTGGACGTT ACATGAATCA GTTCAGTAGA
121     CAAGGCGTAT TCTAATGATA ACAGACAACT TGATAACTCG CATTTCTTCT GAACCCGATG
181     TGATTCAGCA GAACAGTAAA TGTTTCCATG TTTGCTCATC ACTCTCTCTG TGGATTTTTC
241     AGAGACAGAT TTTTTTTTTA TAGTTAGTCT TTGTGAGGCT GACATATTCT GACAAACCCC
301     ATATCCTGGA CGCTTTCCAT AATTTAACTT GATCGTGTCA CTTGTTCGAT CCCCCGCTTG
361     ATAAATATAT AATTCAAAGT GAAACTCTTT CTGGTAAAAA GTGTAGTCTT TAAATCCAAG
421     ATTACTCAGG CCACTTTAGT CATATTTTTC AGATTGACAA ACCTCAGTAT GTAAAATGTG
481     TATAGCCCCT TTAAAACAAG TAAAAACCTT AAGAAAATGA ATAGTAACAG GAGTGCCCCA
541     GTAGGTGGTG TCCCTGGAGG CATTCACCAG CCCTCATCCA ATCTTAGGAC ATGGCATGTA
601     AAATCCTATT CCAGTCAATC TACTTGAGTG GATGGTTAAA AGTAAAAAGC CTTCAATAAA
661     CAGGGGTTAT TATGAAATGC ACTGACATCT GTGTCACCCT GGGGAAAGCC AGTGTGCTAA
721     TAAGTTGAAA GGACTGCCTG AACTCACAGT AGAAAATTAC AAATGGGTAA TATTATTGGA
781     TTATTTTAAT CATTTTATAT GACAAAGTTA AGTATAAGAC ATCGGTATTA AATTGGAACC
841     ATAATCGGAC CACTAACGGG CTGAAAACCA AGTAGCTGCC AGGAACCCTG ACAGGCCCTA
901     TGAAAGATTA ACATATTTCT GACTCACAGC TAAACATTGT TATCAATACT GAGATATGAT
961     CTTACAGAAT GTGGGATGAC GATATGGACA TAAATGATCC ACCTCATTGT TTTATCACTT
1021    CAAATGCTCT GAACAGACCA AAGATAAACT TCATCATTTA GTTTCATGAC TAATATTGGA
1081    AGCGGAAGAT GACACATCAG AAAACAGGCG TTAATTAGAC TTAATTATAG AAACACATTC
1141    CTTACTGTAG ATACATTAGC AGAATAATAA TAAAAAAAAA CCTCTTTGAT GATGATAGCT
1201    ATTGATCATT TCAGCTGGTC ATGTGGGAAA GAATTTCTTA TCTTATCTTA CATTACACGG
1261    GTGGCTAATA TAGAAACGAG TTATGTAAAT ACTGCAGAAA GCCGAGAGAC AAACTAACCC
1321    CTGTGTGTAA TTAAAATCAA GAGCCCACCA TAAATTTGAC TTCTTGTCAT CATTGTATCT
1381    ATACTCATTT TATCATCACA ACCATGCAAA TGAATGGTTT TGCGTGGATT TTCCCTTTAA
1441    ATTATTGGGC TTAAAATATC AGAGGGATTT TTTTTTTTAC ACAATCAGCC TATGTCTATT
1501    TGAAACGTTT GTGTGTGTGT GTGTGTGTGT GTGTGTGTGT GTGTGTGTTA GGAGTTGGGG
1561    CTTATTTATT CTTAAAATAA TCATTTTAAC TTTATCTTCA TTTTAAATAT AAATGAGGAT
1621    AAAGTTCCCG CTGCTTCCTC TGGTCATTTT GAAGATGGAC ACTCCTCGCC ATCTTCATCA
1681    AAATGAATCA CGGATGCTGG ACCGGATGCT TGACGTTTGA TTCCGGGCCT GTCGTAGAGA
1741    GGTATTGGGA AATCCCTCCA GTCTGAAGAA TATAAAATGA AAGCTCATCG CAAAGAACAA
1801    CAACTCCTTC TGACTCCTGA CAGAACCTCA GCATCAGTGG AAACTCAAGT CAAGTGCTTC
1861    CCAGCAGCGA AAC
```

**Supplementary Figure 2.** Sequences of large yellow croaker IL6 promoter cloned. The IL6 promoter plasmid (pGL6-IL6) contains a 1873bp promoter sequence upstream of the IL6 initiation codon(ATG).

A

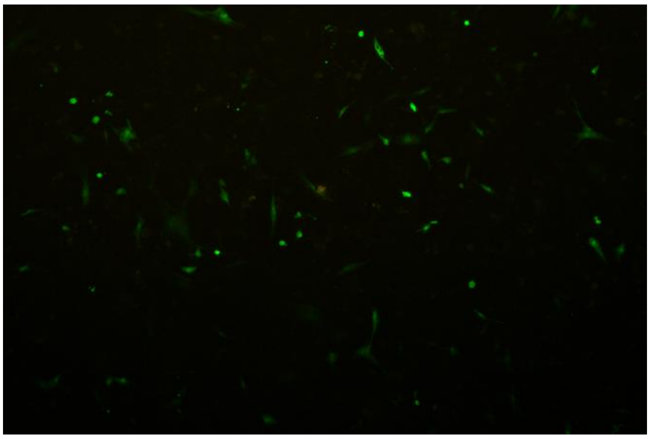

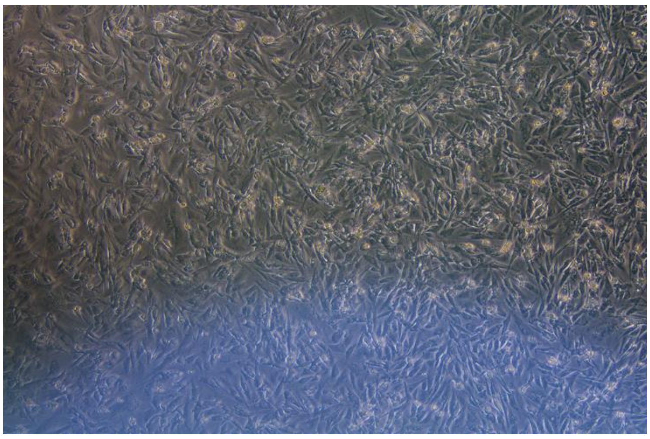

B

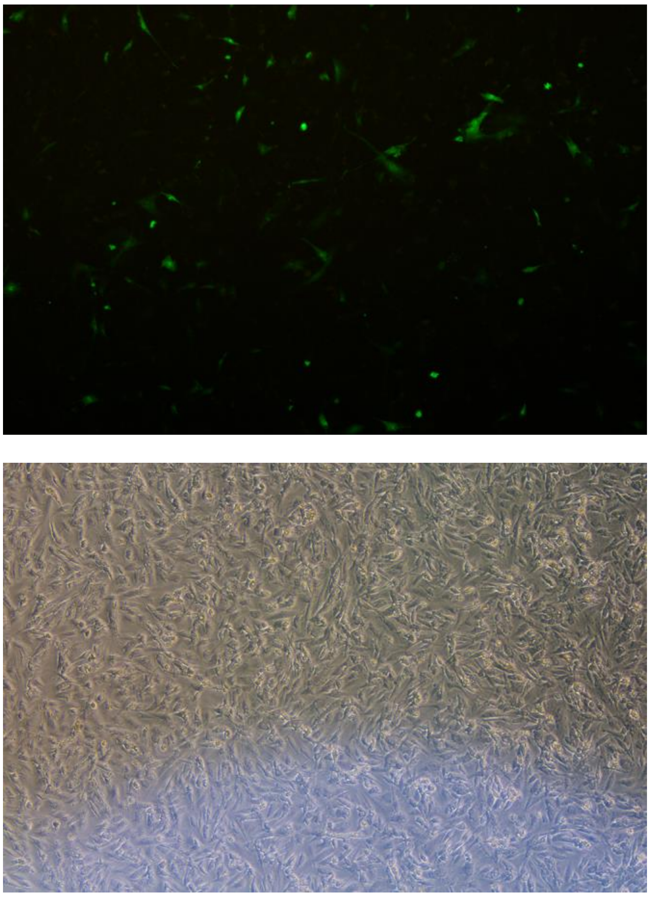

**Supplementary Figure 3.** The overexpression of FFAR4 in large yellow croaker macrophages. After 24h overexpression, the gene expression was observed under a fluorescence microscope. A: pcDNA3.1-EGFP-FFAR4; B: pcDNA3.1-EGFP.
